# RANKL/RANK System-Based Mechanism for Breast Cancer Bone Metastasis and Related Therapeutic Strategies

**DOI:** 10.3389/fcell.2020.00076

**Published:** 2020-02-11

**Authors:** Xiaoqiu Wu, Fangfei Li, Lei Dang, Chao Liang, Aiping Lu, Ge Zhang

**Affiliations:** ^1^Law Sau Fai Institute for Advancing Translational Medicine in Bone and Joint Diseases, School of Chinese Medicine, Hong Kong Baptist University, Kowloon, Hong Kong; ^2^Institute of Integrated Bioinfomedicine and Translational Science, School of Chinese Medicine, Hong Kong Baptist University, Kowloon, Hong Kong; ^3^Institute of Precision Medicine and Innovative Drug Discovery, HKBU Institute of Research and Continuing Education, Shenzhen, China; ^4^Institute of Basic Research in Clinical Medicine, China Academy of Chinese Medical Sciences, Beijing, China; ^5^Institute of Arthritis Research, Shanghai Academy of Chinese Medical Sciences, Shanghai, China

**Keywords:** RANKL, RANK, bone metastasis, breast cancer, vicious cycle, denosumab

## Abstract

Breast cancer remains one of the most life-threatening tumors affecting women. Most patients with advanced breast cancer eventually develop metastatic diseases, which cause significant morbidity and mortality. Approximately two-thirds of patients with advanced breast cancer exhibit osteolytic-type bone metastasis, which seriously reduce the quality of life. Therefore, development of novel therapeutic strategies for treating breast cancer patients with bone metastasis is urgently required. The “seed and soil” theory, which describes the interaction between the circulating breast cancer cells (seeds) and bone microenvironment (soil), is widely accepted as the mechanism underlying metastasis. Disruption of any step in this cycle might have promising anti-metastasis implications. The interaction of receptor activator of nuclear factor-κB ligand (RANKL) and its receptor RANK is fundamental in this vicious cycle and has been shown to be a novel effective therapeutic target. A series of therapeutic strategies have been developed to intervene in this cross-talk. Therefore, in this review, we have systematically introduced the functions of the RANKL/RANK signaling system in breast cancer and discussed related therapeutic strategies.

## Introduction

Breast cancer is one of the most common cancers and the leading cause of mortality in females. The number of patients with breast cancer increases by approximately 1.7 million worldwide each year (Torre et al., 2015). Considering the malignant biological characteristics of breast cancer, local recurrence and distant metastasis are eventually bound to occur. In fact, most of the deaths from breast cancer are not due to the tumor in the primary site, but are the result of distant metastases in the body (Scully et al., 2012; Jin and Mu, 2015). Interestingly, metastasis of breast cancer has organ selectivity, as the lung, liver, and especially the bone are the preferred sites (Fidler, 2003). More than two-thirds of patients with advanced breast cancer eventually harbor osteolytic-type bone metastasis (Suva et al., 2009; Chen et al., 2010). Bone metastasis severely affects the quality of life of patients and indirectly limit life expectancy by inducing bone marrow aplasia, pathological osteolysis, spinal compression, bone fractures, and hypercalcemia, which are referred to as skeletal-related events (SREs) (Yoneda et al., 2013). According to statistics, the average life expectancy of patients with bone metastasis from breast cancer after diagnosis is only 2−3 years (Ibrahim et al., 2013; Cetin et al., 2015).

Most patients with breast cancer exhibit osteolytic lesions; however, osteoblastic or mixed lesions also exist. Bone is a hard tissue with mineralized bone matrix, which renders invasion by cancer cells difficult. Activation of osteoclasts is the central cellular mechanism of osteolytic bone metastasis. Accordingly, breast cancer cells that can migrate and attach to the bone surface must possess special abilities for inducing osteoclastic activation, which then destroy the hard-mineralized bone tissue. Reports show that parathyroid hormone-related protein (PTHrP) can indirectly activate osteoclastogenesis by decreasing the production of osteoprotegerin (OPG) and enhancing the expression of receptor activator of nuclear factor-κB ligand (RANKL) (Boabaid et al., 2004). In addition, the RANKL/RANK signaling system can enhance the proliferation and division of mammary epithelial cells during lobulo-alveolar morphogenesis in an autocrine or paracrine fashion, as well as promote the formation of lactating mammary glands.

Increasing evidence suggests that the RANKL/RANK signaling system is associated with nearly each step in breast cancer development, from primary oncogenesis to the establishment of secondary tumors in the bone. The interaction of breast cancer cells, osteoblasts, osteoclasts, and the bone matrix form a vicious cycle, leading to final bone metastasis. Tumor cells often modulate their gene expression to adapt to the bone microenvironment. For example, most breast cancer cells often express abundant RANK. Simultaneously, the bone also provides a unique nutrient-rich physiological microenvironment to attract tumor cells for migration and colonization in the bone. The bone matrix is rich in various growth factors and cytokines, which provide nutrition to the cancer cells. Breast cancer cells that migrate to bones express cytokines and growth factors such as interleukin (IL)-6, IL-8, IL-11, tumor growth factor (TGF)-β, prostaglandin E, or PTHrP, which can mediate RANKL expression. The up-regulated RANKL activates osteoclastogenesis, following which, many growth factors and cytokines are continually released after the turnover of the bone matrix, which in turn supports cancer cell proliferation and RANKL secretion. In addition to the rich microenvironment of the bone tissue, the abundantly secreted RANKL can also attract RANK-expressing circulating breast cancer cells to migrate into bone matrix, apart from the C-X-C chemokine receptor type (CXCR) 4/12 interaction.

Based on the critical roles of the RANK/RANKL system during the progression of initial primary breast cancer and subsequently bone metastasis, inhibition of RANKL has been developed as a novel therapeutic strategy for advanced breast cancer. Although bisphosphonates have been used for many years, denosumab, the first complete human IgG2 monoclonal antibody for RANKL, has been clinically approved for treating bone-related cancer pathologies and is being used increasingly in clinics (Rizzoli et al., 2010). Similar to OPG, denosumab binds to RANKL and then prevents it from interacting with RANK, thereby decreasing osteoclast differentiation and activation, which consequently reduces bone resorption. Furthermore, denosumab is superior to zoledronic acid in lowering the risk of SREs in patients with metastatic breast cancer and improving their quality of life (Miller, 2009; Stopeck et al., 2010).

## Bone Microenvironment

The establishment of bone metastasis is a complicated process, which involves a vicious cycle consisting of the bone microenvironment and tumor cells. Tumor cells produce adhesive molecules, which enable them to attach to the bone surface. The skeleton is composed of a complex and dynamic tissue controlled by bone-forming osteoblasts and bone-resorptive osteoclasts (Florencio-Silva et al., 2015). During bone remodeling, large quantities of growth factors and cytokines are produced, which then attract the circulating tumor cells (CTCs) to adhere to the bone surface and support metastatic tumor cells to proliferate and survive in the bone tissue (Zheng et al., 2013). Furthermore, the circulating cancer cells that enter the bone can influence bone remodeling and then modulate the bone microenvironment to render it more suitable for tumor cell colonization (Kozlow and Guise, 2005; Akhtari et al., 2008).

### Bone Cells

Single nucleated osteoblasts derived from mesenchymal stem cells (MSCs) play key roles in maintaining bone homeostasis by controlling bone remodeling. Osteoblasts can not only regulate matrix mineralization, but can also affect the expression of extracellular matrix proteins, including type I collagen, osteocalcin, osteopontin, and bone sialoprotein (BSP) (Kini and Nandeesh, 2012). In addition to indirectly stimulating bone resorption by regulating osteoclast differentiation, osteoblasts can also directly attract breast cancer cells by secreting chemotactic factors and adherent molecules. CXCL12 and RANKL, cytokines that are overexpressed by osteoblast-lineage cells and circulate in the blood, can attract CXCR4 and RANK, which are highly expressed on mammary tumor cells, inducing the latter to migrate to the bone surface. A new study regarding prostate cancer bone metastasis demonstrated that prostate cancer cells that produce exosomes can transfer pyruvate kinase M2 (PKM2) into bone marrow stromal cells and up-regulate CXCL12 in these cells (Dai et al., 2019). A similar mechanism remains to be elucidated for breast cancer bone metastasis.

After the synthesis of the new bone matrix, osteoblasts either undergo apoptosis or are embedded in the bone matrix as osteocytes. Evidence showed that the survival of osteocytes is regulated by the osteoprotective factor semaphorin 3A (Sema3A). Estrogen can induce osteocytes to express Sema3A, which acts on the receptors on osteocytes and activates GC-cGMP signaling to promote their survival (Hayashi et al., 2019). Osteocytes can affect bone remodeling by directly communicating with osteoblasts and osteoclasts via mediators that are released in the surrounding medium. For example, RANK secreted by osteocytes primarily regulates osteoclast differentiation and activity. Hence, osteocytes are considered to be mechanical sensors that indicate when and where osteoclasts or osteoblasts absorb or form bone, respectively (Boron and Boulpaep, 2005).

Osteoclasts are large cells with multiple nuclei on the bone surface. These multiple nucleated cells that originate from hematopoietic lines are fused with mononuclear osteoclast precursor cells. Osteoclasts can break down bone tissue via bone resorption (Clines and Guise, 2005). It is well known that only osteoclasts are responsible for absorbing bone (Clarke, 2008). Defects or excessive activity of osteoclasts might result in bone disease such as osteopetrosis or osteoporosis. Christian et al. reported that the iterative fusion of circulating blood monocytic cells with long-lived osteoclast syncytia maintains the function of osteoclasts (Jacome-Galarza et al., 2019). The integrin receptors on the membrane surface osteoclasts assist in binding to the bone matrix peptide; the osteoclasts then secrete acids and lysosomal enzymes to resorb the bone. Osteoclastogenesis is stimulated by two essential factors, macrophage CSF (M-CSF) and RANKL (Teitelbaum and Ross, 2003). Briefly, RANKL binds to the surface of monocytes via RANK. Together with M-CSF, RANKL promotes the fusion of multiple mononuclear cells to form multinucleated osteoclasts. M-CSF activates the signaling pathways via tyrosine kinase Src, resulting in increase in proliferation, survival, and differentiation of osteoclast precursors, which are essential for bone resorption (Clarke, 2008). Both RANKL and M-CSF are not only required for osteoclast formation but are also widely associated with monocyte/macrophage-derived cell differentiation. In addition to relying on RANKL and M-CSF, bone resorption also depends on cathepsin K, secreted by osteoclasts, and hydrogen ions. The function of osteoclasts is regulated by various hormones such as parathyroid hormone (PTH) from the parathyroid gland, which is the main meditator of the vicious cycle leading to bone metastases.

### Extracellular Matrix

During bone destruction and bone formation, different types of growth factors and cytokines are produced and stored in the extracellular matrix (Hauschka et al., 1986; Guise et al., 2005). Almost 95% of the organic bone matrix is composed of collagen type I (Brodsky and Persikov, 2005). The remaining 5% consists of a series of non-collagenous proteins and proteoglycans. During bone formation, osteoblasts secrete bone morphogenetic proteins (BMPs), transforming growth factor beta 1 (TGF-β), insulin-like growth factor (IGF), and fibroblast growth factor (FGF), which are then incorporated into the bone matrix (Chirgwin and Guise, 2000; Guise, 2009). In addition, breast cancer cells can stimulate bone resorption by promoting osteoclastogenesis to release immobilized growth factors.

Growth factors and cytokines released during bone resorption can induce cancer cells to migrate to the bone and support their proliferation. IGF-1 is the most abundant growth factor in the bone extracellular matrix, followed by TGF-β (Crane and Cao, 2014). However, BMPs, FGF1, FGF2, and platelet-derived growth factor (PDGF) are scarce. Evidence shows that only TGF-β plays a direct role in promoting tumor cell metastasis to the bone (Kozlow and Guise, 2005). TGF-β is an important factor that is released during bone resorption, which stimulates tumor cells to express factors such as CTGF, IL-11 (Kang et al., 2003), and PTHrP (Yin et al., 1999), which are necessary for the metastasis of breast cancer cells to the bone. Interestingly, TGF-β mainly mediates growth inhibition at the early stages of tumorigenesis. However, most patients in the advanced stages of the disease lose TGF-β-mediated growth inhibition but still retain TGF-β-mediated regulation of their metastasis-promoting genes (Käkönen et al., 2002; Wakefield and Roberts, 2002). When breast cancer cells colonize the bone environment, molecular mechanisms controlling the interactions among osteoblasts, osteoclasts, hematopoietic stem cells, and other different types of cells are utilized to change bone homeostasis and promote the survival, dormancy, and proliferation of tumor cells (Kan et al., 2016).

## Rankl/Rank/Opg Signaling System

Most breast cancers with bone metastasis are osteolytic, and osteoclasts are considered to be the most effective cells for inducing bone resorption (Charles and Aliprantis, 2014). The RANKL/RANK/OPG system is well-known for regulating bone turnover by promoting the differentiation and activation of osteoclasts (Dougall and Chaisson, 2006; Boyce and Xing, 2007; Silva and Branco, 2011).

### RANKL

RANKL, encoded by tumor necrosis factor superfamily 11 (*TNFSF11*), is also known as osteoprotegerin ligand (OPGL) or osteoclast differentiation factor (ODF) (Hikita et al., 2006). RANKL can be expressed in three different molecular forms: a trimeric transmembrane protein and two different secreted forms arising due to proteolytic cleavage or alternative splicing (Ikeda et al., 2001). RANKL1 is the full-length form of RANKL. RANKL2 is the shorter form of RANKL, which lacks some intra-cytoplasmic domains. RANKL3 is a soluble form of RANKL in which some of the amino acids at the N-terminal are missing. RANKL is highly expressed in various tissues such as lymph nodes, mammary glands, and lung, but is expressed at low levels in the bone marrow and spleen; it is also expressed by osteoblasts, osteocytes, and activated T lymphocytes (Boyce and Xing, 2008). Although RANKL is expressed by many other cells, osteocytes are the main source of RANKL in adult bone tissue (Xiong et al., 2011). In particular, RANKL binds to RANK on myeloid cells, following which it regulates osteoclastogenesis and mediates bone resorption by promoting osteoclast activation and survival (Boyle et al., 2003; Boyce and Xing, 2008). The expression of RANKL in osteoblasts is stimulated by most known factors that stimulate osteoclastogenesis (Udagawa et al., 1999).

Recently, a new study revealed that leucine-rich repeat containing G-protein-coupled receptor 4 (LGR4, also called GPR48) is a novel receptor of RANKL (Luo et al., 2016). The osteoclasts produce LGR4, which directly interacts with RANKL and negatively regulates bone destruction via activation of Gαq/GS3K-β signaling and repression of the NFATc1 pathway during osteoclastogenesis. Mice with *Lgr4* whole body knockout and monocyte conditional knockout exhibited osteoclast hyperactivation and low bone mass. Compared to that in control mice, the number, surface area, and size of osteoclasts dramatically increased in *Lgr4* knockout mice. Furthermore, *in vitro* data showed that LGR4 competes with RANK to bind to RANKL in a dose-dependent manner and suppresses the expression and activity of a series of factors during osteoclastogenesis, attenuating osteoclast differentiation and survival.

OPG, the decoy receptor for RANKL, is secreted mainly by osteoblast lineage cells, and is a potent inhibitor of osteoclast formation, as it competes with RANK for binding to RANKL (Schoppet et al., 2002). Therefore, osteoclast formation is determined by the ratio of RANKL to OPG, rather than by the absolute levels of both molecules (Blair et al., 2006). Systemic factors such as TNF-α, PTH, and interleukins up-regulate the expression of RANKL on osteoblasts relative to that of OPG, resulting in osteoclast activation (Dougall and Chaisson, 2006). A therapeutic strategy with OPG was found to be effective in inhibiting bone resorption for humans (Mundy, 2002).

### RANK

RANK, a type I homotrimeric transmembrane protein, was initially identified only on osteoclast precursors (O), mature osteoclasts, and dendritic cells (Santini et al., 2011). Later, research showed that RANK is also expressed in mammary glands and some other cancer cells with high bone metastasis potential, including breast cancer and prostate cancer (Fata et al., 2000; Chen et al., 2006; Kim et al., 2006). Vesicular RANK secreted by mature osteoclasts can trigger RANKL reverse signaling by binding to RANKL on osteoblasts to promote bone formation (Ikebuchi et al., 2018). On the other hand, RANK on the surface of osteoclast precursors interacts with osteocytic RANKL to promote osteoclastogenesis. The binding of RANKL to RANK results in receptor oligomerization, recruiting TNF receptor-associated factor (TRAF) adaptor proteins (including TRAFs 1, 2, 3, 5, and 6) to specific sites at the membrane-proximal portion of RANK (Armstrong et al., 2002; Akhtari et al., 2008). Unlike other TRAFs, TRAF6, an essential component of the RANKL-RANK signaling pathway, is one of the most critical factors in osteoclast formation and functioning. TRAF6 can activate the c-Jun N-terminal kinase (JNK) and nuclear factor kappa-b (NF-kB) pathways, thereby triggering osteoclastogenesis and bone resorption activity (Wong et al., 1998; Kobayashi et al., 2001). *Traf6* mutant mice exhibited severe osteopetrosis. The relative expression of OPG and RANKL balances this system. Currently, the ratio between RANKL and OPG is considered a therapeutic target for treating estrogen deficiency-associated osteoporosis (Rachner et al., 2011), rheumatoid arthritis (Walsh and Gravallese, 2010), Paget’s disease, and bone tumors.

### Osteoprotegerin (OPG)

Osteoprotegerin, the cytokine receptor of the tumor necrosis factor receptor superfamily encoded by *TNFRSF11B*, is also known as osteoclast formation inhibitor (OCIF). OPG is mainly secreted by osteoblast lineage cells, gastrointestinal epithelial cells, vascular endothelial cells, B cells, and dendritic cells (Sisay et al., 2017). The receptor was originally found to protect bones by preventing bone absorption, as it competes with RANK for binding to RANKL (Hofbauer and Heufelder, 2001). Partial deletion of *TNFRSF11B* resulted in a dramatic increase in RANKL-RANK interactions, which eventually led to juvenile Paget’s disease (Whyte et al., 2002). Mice with OPG mutation show severe bone destruction (Cundy et al., 2002). Furthermore, OPG can also inhibit the apoptosis of human osteoclasts by blocking the TNF-related apoptosis-induced ligand-based signaling system (Chamoux et al., 2008).

## Metastasis of Breast Cancer Cells to the Bone

**FIGURE 1:**
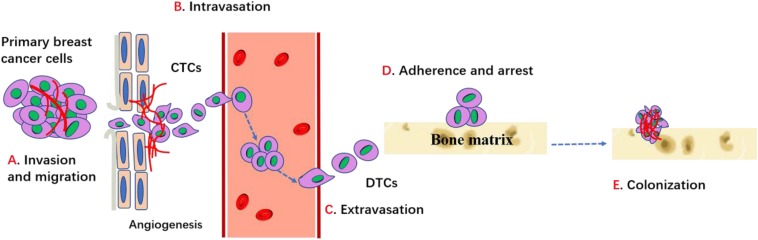
Process of breast cancer bone metastasis. Cancer cells proliferate, invade into surrounding tissues and escape from primary site through the regulation of cadherins, MMPs, integrins and other factors. Then cancer cells enter into the circulation (intravasation) and migrate toward bone through the conduct of chemokines and other factors, etc. When cancer cells egress blood vessels (extravasation) and arrive bone target, it can adhere and bind with bone matrix and get arrest through integrins and cadherin. Then cancer cells can survive, proliferate and differentiate through the interaction with bone microenvironment which ultimately lead to the establishment of bone metastases.

Although osteoblastic and mixed lesions exist, most breast cancer with bone metastasis manifest as osteolytic bone metastasis, which accounts for 80−90% cases. In the case of osteolytic metastases, circulating breast cancer cells not only highly enhance osteoclast activities, but also seriously inhibit osteoblast differentiation and ultimately result in increased bone resorption and decreased bone formation, leading to higher risk of fractures. The metastasis of a primary breast cancer to the bone requires several complicated steps including: (1) proliferation at the primary breast site and acquisition of the corresponding metastatic characteristics; (2) disruption of the basement membrane; (3) invasion of the extracellular matrix; (4) intravascular infiltration and transportation; (5) adherence and retentate (6) migration to the blood vessels to form micro-metastases; (7) colonization of cancer cells and bone destruction (Figure 1). Inability to complete any of these steps will arrest the process (Fidler, 2003).

### Invasion

The primary breast cancer cells first induce angiogenesis to supply nutrients for tumor cell proliferation. Local angiogenesis is necessary for breast cancer cells to reach a clinically detectable size. In addition, the development of new blood vessels also provides the tumor cells a pathway for entering the blood or lymphatic vessels (intravasation). After escaping from the primary site, the invasive breast cancer cells secrete matrix metalloproteinases (MMPs) and cathepsin-K to degrade the ECM and connective tissue and then break through the matrix barrier (Fidler, 2003; Mareel and Leroy, 2003). MMPs are a major class of proteinases necessary for tumor cell invasion. Integrins, the major receptors for extracellular matrix proteins, are also critical for cell invasion and migration. EMT is a process via which epithelioid tumor cells are transformed into mesenchymal fibroids, which show enhanced invasiveness such as mobility and distant metastasis. Evidence suggests that EMT is associated with tumor progression, as it induces proteases such as MMPs that assist in invasion and endosmosis (Bonnomet et al., 2010). Reversion of the invasive breast cancer cells into post-mitotic adipocytes may repress primary tumor invasion (Ishay-Ronen et al., 2019). Experimental results showed that high level of RANK in human mammary epithelial cells can induce EMT and increase migration (Palafox et al., 2012). Primary breast cancer cells must successfully exudate through the bone matrix to enter systemic circulation.

### Migration

The breast cancer cells that enter the systemic circulation survive by evading both the host’s immune response and apoptotic signals (Valastyan and Weinberg, 2011). Tumor cells that enter the systemic circulation are called circulating tumor cells (CTCs), which are disseminated to the bone through the bloodstream or lymphatic vessels. Adhesion to the vascular endothelial cells is the key for the escape of CTCs from the blood vessels. After entering the bone, the CTCs are exposed to factors in the bone microenvironment that support the growth and egress circulation (extravasation) of metastasized cells and promote colonization in the bone. These disseminated cancer cells are called disseminated tumor cells (DTCs). DTCs readily change their biological phenotype to adjust to bone environments and acquire new malignant capacities (Lambert et al., 2017). For this targeted migration, the bone also produces numerous cytokines and chemokines that attract primary tumor cells and induce them to home to the bone (Mastro et al., 2003). Human breast cancer cells express more CXCR4/RANK than normal epithelial cells, and CXCL12, the receptor of CXCR4, is highly expressed in the liver, lung, and bone marrow. Furthermore, breast cancer cells produce factors such as PTH or PTHrP, IL-1, and IL-6, which stimulate osteoblasts and stromal cells to secrete RANKL. The interactions between CXCR4 and CXCL12, RANKL, and RANK are critical for the homing of breast cancer cells to the bone marrow (Müller et al., 2001; Sun et al., 2005).

### Adhesion and Survival

Adhesion is the next step after the breast cancer cells have migrated to the bone. Integrins and cadherins are major regulators for this specific adhesion. Integrin is an isodiglycan protein that mediates the interaction between breast cancer cells and the extracellular matrix. Arg-gly-asp (RGD), a tripeptide binding to αvβ3 integrin, is one of the extracellular matrix proteins present in vitronectin, osteopontin, and osteosialin, which mediates migration of tumor cells to the trabecular bone. Evidence suggests that hemocyte adhesion in the blood vessels is dependent on and is regulated by integrin activation (Felding-Habermann et al., 2001). Breast cancer cells that overexpress αvβ3 integrin are more prone to bone metastasis (Pecheur et al., 2002). The interaction of *E*-cadherin derived from tumor cells and *N*-cadherin derived from osteogenic cells activates the mTOR signaling pathway, which mediates the binding of these cancer cells to bone marrow stromal cells and then promotes homing of the breast cancer cells to the bone marrow (Wang et al., 2015).

Survival and proliferation in the bone are the next key steps underlining the success of bone metastasis after the tumor cells have adhered to the bone tissue. The metastatic breast cancer cells overexpress a series of factors such as PTHrP to stimulate osteoblasts to highly express RANKL and reduce OPG expression. Therefore, bone resorption is promoted, which results in the release of growth factors, including BMPs, TGF-β, IGFs, FGFs, PDGFs, and calcium. These growth factors may not directly promote cancer cell proliferation but may indirectly induce angiogenesis and osteoclastogenesis to remodel the bone microenvironment for tumor cell colonization. In particular, the abundant calcium in the bone matrix can attract the calcium-sensing receptor-expressing breast cancer cells to the bone. Furthermore, Wang et al. (2018) showed that the increased intracellular calcium concentration in metastatic breast cancer cells induced by connexin 43 can disturb the osteogenic niche and promote survival (Waning et al., 2019). Furthermore, the bone microenvironment promotes breast cancer cells to transit to a migratory phenotype with new malignant capacities. For instance, compared to that in the orthotopic mammary fat pad of mice, the increased expression of Snail, a well-recognized mesenchymal marker, and the decreased expression of *E*-cadherin, a representative epithelial marker, were observed in the bone metastatic breast cancer cells, suggesting that the bone microenvironment promotes EMT of bone metastatic tumor cells. Further evidence showed that EMT is induced by TGF-β secreted during bone resorption (Heldin et al., 2012). Supported by the bone microenvironment, tumor cells successfully colonize the bone.

## The Rankl/Rank System Is Involved in Osteoclast Differentiation and Activation

Osteoclasts are key participants in bone remodeling, as they regulate bone structure and function in adulthood. Recently, scientists have made new breakthroughs in understanding the mechanisms underlying the differentiation and activation of osteoclasts, and the RANKL/RANK signaling system has been identified as the key regulator of osteoclastgenesis.

**FIGURE 2:**
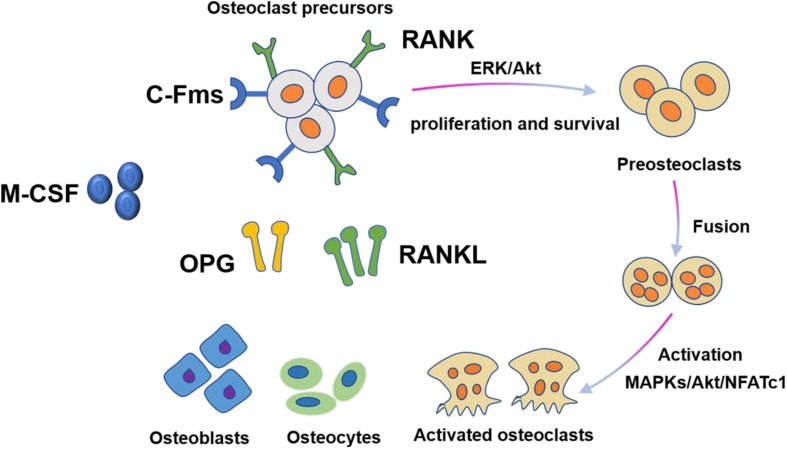
Osteoclast differentiation is stimulated by M-CSF and RANKL. M-CSF induces the proliferation and survival of osteoclast precursor cells through activation of ERK and Akt. RANKL recruits TRAF6 to activate MAPKs, Akt, and NFATc1 to promote differentiation of osteoclast precursors to osteoclasts.

M-CSF and RANKL are two essential cytokines that regulate differentiation of hematopoietic lines into osteoclasts (Figure 2). During this process, M-CSF acts as a survival factor of early progenitor cells, whereas the RANKL-RANK signaling system acts as critical instructive osteoclast lineage signal (Nagy and Penninger, 2015). Similar to RANKL, M-CSF also originates from bone marrow stromal cells. M-CSF was originally identified to be necessary for promoting the survival of osteoclast progenitor cells and was essential for osteoclast development (Boyce and Xing, 2007). A mouse model expressing non-functional M-CSF develops osteoclast-deficient osteopetrosis, which was indicative of the key role of M-CSF in osteoclastogenesis. The interaction of M-CSF and c-FMS leads to the dimerization and phosphorylation of c-FMS, which then provides necessary signals for the proliferation and survival of osteoclast precursor cells. However, M-CSF by itself cannot complete this process. The differentiation of osteoclast precursor cells also require RANKL expression from osteoclast stromal cells and RANK expression from osteoclast precursor cells (Boyle et al., 2003). In the presence of M-CSF, RANKL binds to RANK and then activates transcription factors such as NF-κB, promoting the differentiation of osteoclast progenitors and limiting the apoptosis of mature osteoclasts. In addition to activating multiple transcription factors, RANKL also promotes osteoclast formation by stimulating all three families of mitogen-activated protein (MAP) kinases (Wada et al., 2006). Importantly, the activation of the activator protein 1 (AP-1)/nuclear factor of activated T-cells cytoplasmic 1 (NFATc1) transcription complex is a major osteoclastogenic event. RANKL produces this transcription complex by increasing the expression of the c-Fos family of proteins and promoting the nuclear translocation of Jun proteins. The dephosphorylation of NFATc1 by calcineurin in turn promotes its nuclear translocation. Dysfunction of c-Jun, c-Fos, or NFATc1 can lead to failure of osteoclast activation and cause severe osteopetrosis. Furthermore, RANKL can also promote bone destruction by inducing mature osteoclasts to generate a complex consisting of their receptor TRAF6 and c-Src, which specifically recruit cytokines to lipid rafts in the plasma membrane (Teitelbaum and Ross, 2003).

## Functions of the Rankl/Rank System in Primary Breast Cancer Development and Bone Metastasis

To successfully survive in the primary and secondary site, breast cancer cells must possess certain unique characteristics and the bone matrix needs to provide a suitable environment.

### The RANKL/RANK System Is Involved in the Initial Phases of Breast Cancer Development

In addition to the essential roles of the RANKL/RANK system in bone metabolism, reports show that RANK and RANKL act as key factors in the formation of lactating mammary glands during pregnancy (Fata et al., 2000). The expression of RANKL on mammary epithelial cells is induced by sex and pregnancy hormones. RANKL deficiency can affect the formation of the lobo-alveolar structures, which is required for lactation. RANKL expression is required for promoting the survival and proliferation of epithelial cells, as well as for lobulo-alveolar development. The dysregulation of this coordinated mechanism in transgenic mice might promote the formation of pre-neoplasias and subsequently tumor foci (Gonzalez-Suarez et al., 2010). In addition, the RANKL/RANK axis is pro-active in EMT; therefore, the RANKL/RANK system also mediates the expansion of mammary stem cells and controls the development of hormone-dependent breast cancer. Reduction in RANK expression in the mammary gland of female mice can dramatically decrease sex hormone-induced mammary cancer. Furthermore, RANK/RANKL confers resistance to γ-irradiation-induced cell death in mammary epithelial cells, changes cell adhesion, and regulates the self-renewal capacity of tumor stem cells, contributing to the development of mammary cancer.

### The RANKL/RANK System Contributes to Bone Metastasis

#### Direct Mechanisms

In addition to the chemotaxis caused by the interaction of CXCR4/12, RANKL in bone tissue can also attract RANK-expressing circulating breast cancer cells to the bone matrix (Jones et al., 2006; Jacob et al., 2011). Jones et al. (2006) was the first to demonstrate the chemoattractant activity of RANKL (Chawla et al., 2013). They demonstrated that the osteoblasts and bone marrow stromal cells that produced RANKL could not only attract RANK-expressing tumor cells, but also induce their migration. This mechanism is widely accepted and has been observed in many other types of cancer, including breast cancer. Current evidence suggests that around 25% breast cancer cells express RANK. Cells overexpressing RANK in intra-arterial injection showed faster tumor progression and lower overall survival rate due to enhanced bone homing and colonization than wild-type MDA-MB 231 cells. *In vitro* studies have shown that RANK overexpression in MCF-10A cells decreases intercellular contact and enhances migration (Palafox et al., 2012). RANKL-enhanced migration activates specific signaling cascades such as the MAP kinase pathways. Subsequently, the RANKL/RANK axis regulates the migration of breast cancer cells, and RANKL acts as a chemo-attractive agent on tumor cells overexpressing one of its receptors. *In vivo*, blocking of this signaling with AMG161 (IgG1 equivalent to denosumab) reduces the formation of bone marrow micro-metastasis (Sousa et al., 2018).

#### Indirect Mechanisms

In addition to directly attracting RANK-expressing breast cancer cells, RANKL can also modulate the microenvironment of the metastatic site. RANKL is not only expressed by the mammary epithelial cells but is also expressed on the surface of breast cancer cells. RANKL can promote both the survival and proliferation of epithelial cell precursors during breast development and simultaneously up-regulate the expression of RANK. By interacting with RANK, RANKL can facilitate neoformation of vascular tubes and then stimulate angiogenesis via the Src and phospholipase C-dependent pathway. Blood vessels provide necessary nutrients for cancer cell proliferation, and also act as one of the most prevalent means of cell migration. In addition, RANKL increases vascular permeability, which assists the breast tumor cells in escaping from the blood vessels into systematic circulation.

## The Vicious Cycle of Bone Metastasis

More than a century ago, Stephen Paget first presented the “seed and soil” theory to describe the relationship between CTCs (seed) and metastatic sites (soil) (Langley and Fidler, 2011). Tumor cells of varying origin prefer to metastasize to different organs (Ben-Baruch, 2009). The bone is the most preferred organ for breast cancer metastasis. Although osteoblastic or mixed lesions have been observed, most breast cancer metastasis are bone lytic diseases. Osteolytic bone metastasis accounts for 80−90% cases among all patients with metastatic breast cancer (Kang et al., 2003). The exact mechanism underlying the migration of breast cancer cells to the bone remains to be elucidated, although current evidence indicates that bone metastasis is related to a crosstalk between breast cancer cells and the bone microenvironment (Azim and Azim, 2013).

Owing to the combined efforts of chemokines, circulating breast cancer cells are attracted to the bone (Sun et al., 2010). The mineralized bone matrix releases relevant growth factors, including IGFs, TGF-β, FGFs, PDGFs, and BMPs, when interacting with metastatic breast cancer cells, and therefore, it offers a specific fertile environment for tumor cell proliferation and aggression. PDGFs, BMPs, and calcium in the bone matrix can enhance the survival of tumor cells (Sanders et al., 2000; Weilbaecher et al., 2011). Experimental evidence showed that bone-derived IGF can promote the proliferation and survival of cancer cells via the Akt/NF-κB pathway (Samani et al., 2007; Maki, 2010). As the second-most abundant growth factor in the bone extracellular matrix, TGF-β plays critical roles in inducing the vicious cycle.

**FIGURE 3:**
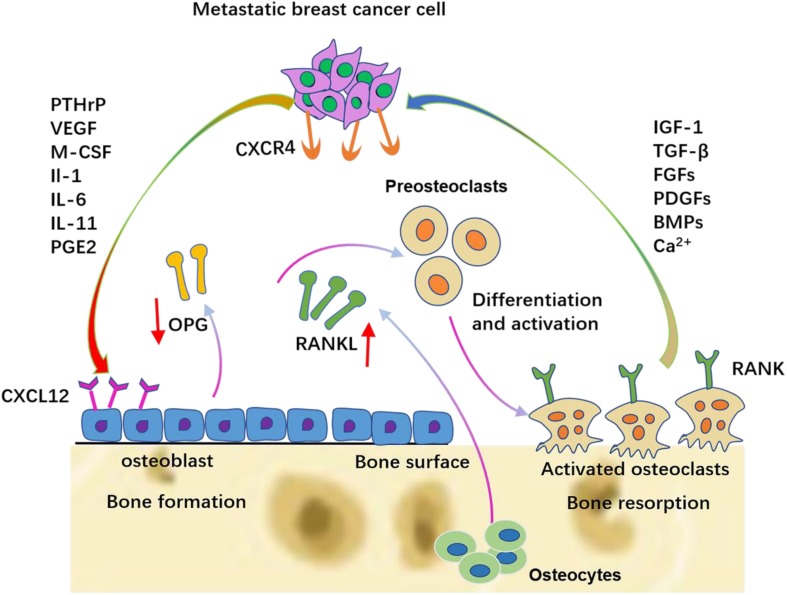
The vicious cycle between breast cancer cells and bone. Breast cancer cells secrete soluble factors, including PTHrP, VEGF, IL-6, IL-8, and IL-11, which in the bone metastatic site act on osteoblasts/osteoclasts. The production of RANKL is increased and the production of OPG is decreased from osteoblasts. Late-stage pre-osteoclast cells respond to specific breast cancer-secreted factors by differentiation and osteolytic activation.

After the colonization of DTCs, bone-derived TGF-β stimulates breast cancer cells to synthesize PTHrP, prostaglandin E2 (PGE2), IL-1, IL-6, and IL-11. PTHrP is one of the most important factors in mediating osteoclast activation in metastatic human breast cancer. Breast cancer cells present in the metastatic bone site produce higher amounts of PTHrP than cells in the breast or soft-tissue sites (Soki et al., 2012). These cytokines first bind to osteoblasts or stromal cells via the corresponding receptors and then up-regulate the expression of RANKL and simultaneously down-regulate the expression of OPG. The up-regulated RANKL interacts with RANK on the hematopoietic osteoclast precursors to induce mature osteoclast production. Therefore, these cytokines stimulate osteoclastic bone destruction indirectly. The resorbed bone can further release TGF-β and IGF1, thereby stimulating the proliferation of tumor cells to produce more PTHrP via Smad and the p38 MAPK signaling pathway (Le Pape et al., 2016), which in turn causes more bone resorption. This is termed the vicious cycle (Figure 3).

## Rankl/Rank System-Based Therapeutic Strategies for Breast Cancer Bone Metastasis

Metastatic cancer cells must possess the ability to promote bone resorption to establish and grow in bone tissue. Hence, the key factors that prevent or hinder metastasis must reduce osteoclast differentiation and activation. Therefore, regulation of the main factors involved in the metastatic process, especially in the vicious cycle, should be promising.

### Bisphosphonates

Currently, bisphosphonates are clinically used to treat bone complications of malignant tumors. Bisphosphonates, which include amino-bisphosphonates (ibandronate, pamidronate, and zoledronic acid) and non-amino bisphosphonates (clondronate), hinder the survival and stimulate apoptosis of mature bone-resorbing osteoclasts to suppress bone resorption. However, bisphosphonates act only on the mature, actively resorting osteoclasts, but has no effect on the residual osteoclasts (Morony et al., 2005). This might explain why zoledronic acid, which has been approved by the Food and Drug Administration (FDA) to treat patients with solid tumor bone metastasis, does not affect the patient survival rate (Coleman, 2002, 2004). Furthermore, many patients continue to develop SREs after bisphosphonate treatment, which warrants development of novel and effective strategies for managing these patients.

### Direct and Indirect Targeting of RANKL

As previously mentioned, RANKL plays fundamental roles in the progression of primary breast cancer and bone metastasis. Hence, targeting RANKL has been an important therapeutic approach for patients with osteoclastic bone metastasis.

As OPG is one of the receptors of RANKL, interfering with RANKL function might be an efficient way of decreasing bone destruction (Morony et al., 2005). Canon et al. showed that administration of an OPG-Fc construct in a mouse model with breast cancer bone metastasis may block skeletal tumor progression and improve survival by inhibiting RANKL (Canon et al., 2008). Therefore, a genetically modified recombinant OPG-Fc construct (AMGN-0007) was developed for treating patients with bone metastases. When used for treating patients with breast cancer or multiple myeloma, AMGN-0007 has the same effect as pamidronate in reducing bone destruction (Body et al., 2003). Nevertheless, the short half-lives of several factors have prevented further research and application of this drug in clinical use. In addition, OPG can not only bind to RANKL, but can also block TNF-related apoptosis inducing ligand (TRAIL) (Emery et al., 1998). Importantly, TRAIL is considered to be a significant part of natural anticancer immunity and is the main mediator for host immune cells in inducing tumor cell death (Neville-Webbe et al., 2004).

On the other hand, a monoclonal antibody specific to human RANKL, denosumab, has shown promising clinical implications (Cummings et al., 2009). In addition, evidence shows that denosumab is efficient and safe, and is better than zoledronic acid in delaying or preventing SREs (Stopeck et al., 2010; Martin et al., 2012). Currently, denosumab has been approved by the FDA for treating treatment-induced bone loss and osteoporosis in women with high risk of fractures and metastases to bone. Denosumab acts by binding to human RANKL with high affinity and specificity and blocks its binding to RANK, thereby inhibiting RANK-medicated osteoclast formation, ultimately increasing bone volume and density (Cummings et al., 2009). Unlike OPG, denosumab not only avoids potential reactions with TRAIL, but also exerts the same beneficial effects as OPG (Abrahamsen and Teng, 2005). Denosumab is administered via subcutaneous injection and cannot be excreted via the kidney or be metabolized by the liver owing to its large molecular weight. Furthermore, unlike the mechanism of action of bisphosphonates, denosumab blocks the differentiation, activation, and survival of osteoclast precursor cells and completely eliminates osteoclasts in the treated bone tissue (Baron et al., 2011). Some clinical trials involving denosumab are shown in Table 1.

**TABLE 1:** Some clinical trials which are involved in denosumab.

**Status**	**Study title**	**Goal**	**Study phase**	**Clinical trial Gov. Identifier**
Completed	Denosumab for Breast Cancer with Bone Mets	To learn if denosumab in combination with a hormonal drug can help lower the number of circulating tumor cells (CTCs) in patients with breast cancer that has spread to the bone.	Phase 2	NCT01952054
Terminated	Study of Denosumab as Adjuvant Treatment for Women with High Risk Early Breast Cancer Receiving Neoadjuvant or Adjuvant Therapy (D-CARE)	To study the effect of denosumab to see if it can prevent disease recurrence in the bone or in any other part of the body, when it is given as adjuvant therapy for women with early-stage breast cancer, who are at high risk of disease recurrence.	Phase 3	NCT01077154
Terminated	A Study to Evaluate Denosumab in Young Patients with Primary Breast Cancer	To determine if a short course of RANKL inhibition with denosumab can induce a decrease in tumor proliferation rates as determined by Ki67 immunohistochemistry (IHC) in newly diagnosed, early stage breast cancer in pre-menopausal women.	Phase 2a	NCT01864798
Completed	Denosumab (AMG 162) in Bisphosphonate Naive Metastatic Breast Cancer	To evaluate various doses and schedules for denosumab administration and characterize the safety profile in this indication.	Phase 2	NCT00091832
Completed	AMG 162 in the Treatment of Bone Loss in Subjects Undergoing Aromatase Inhibitor Therapy for Non-metastatic Breast Cancer	To evaluate AMG 162 in the treatment of bone loss in subjects undergoing Aromatase Inhibitor Therapy for Non-metastatic Breast Cancer.	Phase 3	NCT00089661
Terminated	Denosumab in Treating Patients with ER and/or PR Positive, HER2 Negative Metastatic Breast Cancer With Bone Metastases and Detectable Circulating Tumor Cells	To look at the amount of cancer cells in the blood of participants who are being treated with denosumab. To look at how long it takes for cancer to get worse when participants are being treated with denosumab.	Phase 2	NCT03070002
Completed	A Study Comparing Denosumab vs. Zoledronic Acid for the Treatment of Bone Metastases in Breast Cancer Subjects.	To determine if denosumab is non-inferior to zoledronic acid in the treatment of bone metastases in subjects with advanced breast cancer.	Phase 3	NCT00321464
Completed	RANKL Inhibition and Breast Tissue Biomarkers	To investigate the effect of RANKL inhibition with denosumab on breast tissue markers in high-risk premenopausal women with dense breasts.	Phase 1	NCT03629717
Completed	Open Label Extension to SRE Studies in United Kingdom and Czech Republic Only	To describe the safety and tolerability of denosumab administration as measured by adverse events, immunogenicity, and safety laboratory parameters in subjects who previously received either zoledronic acid (Zometa) or denosumab.	Phase 3	NCT00950911
Completed	Study of AMG 162 in Subjects with Advanced Cancer Currently Being Treated with Intravenous (IV) Bisphosphonates	To determine the effectiveness of AMG 162 in reducing urinary N-telopeptide in advanced cancer subjects with bone metastases.	Phase 2	NCT00104650
Completed	Study of Denosumab in the Treatment of Hypercalcemia of Malignancy in Subjects with Elevated Serum Calcium	To determine the potential of denosumab to treat Hypercalcemia of Malignancy in patients with elevated serum calcium who do not respond to recent treatment with intravenous bisphosphonates by lowering corrected serum calcium < / = 11.5 mg/dL (2.9 millimoles/L) by day 10.	Phase 2	NCT00896454

It is noteworthy that three clinical trials were approved for determining the effectiveness of denosumab in order to prevent the skeletal sequelae in patients with bone metastases. These clinical trials showed that denosumab caused more severe hypocalcemia than zoledronic acid (Lipton et al., 2010). Medication-related osteonecrosis of the jaw (MRONJ) involves progressive bone destruction in the jaws that occurs when anti-resorptive or anti-angiogenic drugs are used. The first case of denosumab-related ONJ was reported in a 73-year-old man diagnosed with prostatic adenocarcinoma. These clinical trials demonstrated that events of ONJ occurred more in the denosumab group than in the zoledronic acid group (2.0%, denosumab; 1.4%, zoledronic acid) (Saad et al., 2011). Therefore, whether denosumab is the best therapeutic choice for patients should be evaluated.

## Conclusion

The common cause of death for breast cancer patients is bone metastasis, nearly 80% of which are due to osteolytic lesions. Bone metastasis always leads to devastating consequences. The life expectancy for patients with metastatic breast cancer is 2−3 years. Bone metastasis also deteriorates the quality of life by inducing severe bone pain and SREs. Therefore, the mechanism of osteolysis should be understood to design relevant therapeutic strategies. In this article, the roles of the RANKL/RANK axis in the development of primary breast cancer and secondary establishment of bone metastasis were reviewed. The steps involved in the development of bone metastasis were also clearly described.

The RANKL/RANK system plays a critical role in breast cancer development, both during initial tumorigenesis and formation of secondary tumors in the bone. RANKL can promote the survival and proliferation of epithelial cells during the development of mammary glands and simultaneously up-regulate the expression of RANK. The RANKL/RANK system is pro-active in EMT and can promote cell migration and neovascularization (Min et al., 2003). In addition, similar to the CXCR4/12 interaction, RANKL can also attract circulating breast cancer cells to the bone matrix via the RANKL/RANK system. Therefore, targeting of RANKL has been a novel therapeutic target for treating breast cancer bone metastasis.

Currently, bone metastasis of primary breast tumors appears incurable, and disruption of the osteolytic cycle by targeting osteoclasts constitutes the main therapeutic intervention. Bisphosphonates have been used to treat patients with breast cancer bone metastasis, albeit severe side effects. Therefore, more effective therapies are urgently required. The functions of RANKL in breast cancer metastasis to bone have been described in detail in this review. Denosumab, a completely human IgG2 monoclonal antibody, was used to treat patients with osteoporosis, metastases to bone, and treatment-induced bone loss. Denosumab competitively binds to RANKL, thereby blocking the binding of RANKL to RANK and inhibiting RANK-medicated osteoclastogenesis, ultimately increasing bone volume and density. The main side effect of denosumab might be the incidence of osteonecrosis of the jaw in few patients. In addition, denosumab has no effect on the survival rate of patients with bone metastasis, which suggests that inhibition of osteoclastic resorption alone might be not sufficient for suppressing tumor dissemination. Further research is required to understand the mechanism of metastasis and develop more effective therapies directed at improving the quality of life of patients with bone metastasis.

## Author Contributions

XW wrote the manuscript. FL and LD helped in revising the manuscript. XW contributed in figure designing. CL contributed to the manuscript for literature research. AL and GZ revised and approved the manuscript.

## Conflict of Interest

The authors declare that the research was conducted in the absence of any commercial or financial relationships that could be construed as a potential conflict of interest.

## Abbreviations

AP-1: Activator protein 1
BSP: Bone sialoprotein
c-FMS: Colony-stimulating factor 1 receptor
c-JNK: c-junN-terminal kinase
CTCs: Circulating tumor cells
CTGF: Connective tissue growth factor
CXCR 4/12: C-X -C chemokine receptor type 4/12
DTCs: Disseminated tumor cells
EMT: epithelial mesenchymal transition
FGF: Fibroblast growth factor
IGF: Insulin-like growth factor
IL-6: Interleukin 6
LGR4: G-protein -coupled receptor 4
MAPK: Mitogen-activated protein kinase
M-CSF: Macrophage CSF
MMPs: Matrix metalloproteinases
MSC: mesenchymal stem cell
NFATc1: Nuclear factor of activated T-cells cytoplasmic 1
NF-kB: Nuclear factor kappa-b
OCIF: Osteoclast formation inhibitor
OCPs: Osteoclast precursors
ODF: Osteoclast differentiation factor
ONJ: Osteonecrosis of the jaw
OPG: osteoprotegerin
OPGL: Osteoprotegerin ligand
PDGFs: Platelet-derived growth factors
PGE2: Prostaglandin E2
PTH: Parathyroid hormone
PTHrP: Parathyroid hormone-related protein
RANK: Receptor activator of nuclear factor- κ B
RANKL: Receptor activator of nuclear factor- κ B ligand
Sema3A: osteoprotective factor semaphorin 3A
SREs: skeletal-related events
TGF-β: Transforming growth factor beta 1
TNFSF11: Tumor necrosis ligand superfamily member 11
TRAF: TNF receptor-associated factor
TRAIL: TNF related apoptosis inducing ligand
TRANCE: TNF-related activation induced cytokine.
